# An overview of treatment for temporomandibular disc displacement including disc reduction

**DOI:** 10.22514/jofph.2025.024

**Published:** 2025-06-12

**Authors:** Jingyu Ye

**Affiliations:** ^1^School of Stomatology, Zhejiang Chinese Medical University, 310053 Hangzhou, Zhejiang, China; ^2^Oral Sciences Research Group, Glasgow Dental School, School of Medicine, Dentistry and Nursing, College of Medical, Veterinary and Life Sciences, University of Glasgow, G2 3JZ Glasgow, UK

**Keywords:** TMJ, Anterior disc displacement, Treatment, Disc reduction

## Abstract

This article reviews the various treatment options for anterior temporomandibular joint disc displacement, including disc reduction. These options include functional splints, anchorage, arthroscopy, combined orthodontic and disc repositioning, and combined orthognathic and disc repositioning. This article summarizes and analyses the indications, advantages, disadvantages, clinical efficacy, and available supportive trials of each treatment option. The objective of this study is to guide clinicians in the fields of temporomandibular joint orthognathic and orthodontics in their multidisciplinary diagnosis and combined treatment planning. Additionally, the article serves as a comprehensive reference for the selection of treatment options for clinicians. Conservative treatments can provide symptomatic relief, but it is difficult to reposition the disc. Arthroscopy or anchorage techniques can effectively reposition disc displacement, laying the foundation for subsequent treatment. The use of conservative treatments after surgery, combined with orthodontic and orthognathic treatment, can facilitate the promotion of effects and the prevention of recurrence. However, further research is needed to investigate long-term outcome, such as the impact of early intervention in adolescents on facial shape development.

## 1. Introduction

### 1.1 Etiology and epidemiology

Temporomandibular joint disorder (TMD) is a common condition in stomatology, alongside caries, periodontal disease, and malocclusion. TMD encompasses a spectrum of conditions affecting the temporomandibular joint (TMJ), masticatory muscles, nerves, and other associated structures. TMDs primarily affect young and middle-aged individuals, with the highest prevalence and consultation rates in the age group of 20–30 years. A significant increase in the prevalence of TMD has been observed. The prevalence of TMD ranges from approximately 5% to 12% in the general population and between 7.3% and 30.4% in adolescents aged 10–19 years [1, 2]. The cause of the disease is uncertain, but it is believed to be linked to various factors, such as psychosomatic, occlusal, immunologic, joint overloading, and anatomical factors. Some scholars also suggest a relationship between TMD and hormone levels [3].

### 1.2 Clinical symptoms, diagnosis and staging

Symptoms of TMD commonly include pain during temporomandibular joint movement, clicking and murmuring. Additionally, related pain in the masticatory muscles, headaches on the affected side, limited mouth opening, and other related mandibular movement abnormalities and dysfunctions are related to TMD. According to the diagnostic criteria established by Schiffman, TMD can be classified into 12 common diagnoses, including arthralgia, myalgia, local myalgia, myofascial pain, myofascial pain with referral, four-disc displacement disorders, degenerative joint disease, subluxation, and headache attributed to TMD [1]. The prevalence of anterior disc displacement (ADD) is 41.1%, making it the most common type of TMD in community samples and involving abnormal disc-condyle relationships [4]. ADD can be classified into two categories: anterior disc displacement with reduction (ADDwR) and anterior disc displacement without reduction (ADDwoR). In the latter category, the disc is in an anterior position relative to the condyle when the mouth is closed and reduction is not possible when the mouth is opened [5].

In 1989, Wilkes proposed a staging system that can provide a diagnostic protocol for various stages of internal derangement (ID) of the TMJ on the basis of clinical, radiological, and surgical examinations [6]. Despite the widespread adoption of the Wilkes staging system in the field of temporomandibular joint disease, there remains a paucity of consensus regarding the optimal treatment approach for patients at different stages. In 2019, Yang’s staging system was established, aiming to provide a reasonable treatment protocol for ADD patients with varying severity. The new staging system appears to be a reliable and beneficial tool for the planning and prediction of patient prognosis and treatment [7].

### 1.3 Imaging method

In recent years, significant advances in medical imaging have greatly facilitated the study of TMD. The diagnosis of disc displacement can be made on the basis of an examination of the relevant clinical symptoms and imaging procedures [8]. Magnetic resonance imaging (MRI) is considered the gold standard for evaluating soft tissues, including the articular disc. MRI provides information about the position, morphology, and structure of the disc, the quantity of synovial fluid, the condition of the bone, the posterior attachment, and the retrodiscal tissues [9]. Computed tomography (CT) is primarily used to diagnose bone lesions, including bone erosion, fractures, postoperative deformities, and deformities of the adjacent temporal bone [10]. Bone scanning is useful for evaluating osteoarthritis and joint inflammation [11]. Ultrasound is not limited to disc displacement but is also useful for assessing degenerative changes to joint surfaces, joint effusion, and synovitis [12]. Although conventional radiographs, such as panoramic and cephalometric radiographs, have low accuracy in detecting temporomandibular joint disease [13], they can help clinicians quickly overview patients’ oral basics.

### 1.4 Treatment and risk

Various treatments are available to treat ADD, ranging from non-invasive approaches, such as conservative treatments, and functional splints, including anterior repositioning splints (ARS), stabilization splints (SS), and other innovative splints, to more invasive surgical repositioning methods, such as open disc repositioning (ODR), arthroscopic disc repositioning (ADR), and other combined therapies.

In 1887, Annandale published the first description of surgical repositioning of the TMJ disc, and ARS was first described by Farrar in the 1970s [14, 15]. In 1979, McCarty and Farrar described the procedure of TMJ internal derangement by ODR to reposition the disc to the normal disc-condyle relationship [16]. In 1975, Onishi developed arthroscopy by using an arthroscope in the temporomandibular joint for the first time, and a new method for the diagnosis and treatment of the temporomandibular joint was developed [17, 18]. With advancements in arthroscopic techniques and equipment, the ADR procedure was developed to reposition and correct disc-condyle relationships in a minimally invasive manner [19]. Over the last two decades, there has been an increase in innovative and combination therapies that have improved upon previous treatments.

However, the immediate and long-term effects of these treatments remain uncertain. Conservative treatment methods, such as functional splints, may cause patient discomfort and difficulty maintaining oral hygiene, leading to caries, periodontal diseases, or candidiasis [20]. Futhermore, the percentage of joint redisplacement is estimated to range from 27.47% to 59.4% [21]. Surgical treatment methods can result in local swelling, pain, numbness, and facial paralysis, among other risks, accounting for an overall complication rate of 7.71% [22]. Surgery is also more traumatic and expensive and requires a highly skilled personnel.

### 1.5 Clinical significance of this review

In recent years, interest in the relationships among ADD, condylar resorption, and dentomaxillofacial deformities has grown [23, 24, 25, 26]. Persistent ADD can interfere with condyle development, especially in teenagers [27]. Therefore, ADD during adolescence can significantly affect a patient’s facial appearance, oral function, and mental health [28]. Additionally, unilateral ADD in teenagers can cause asymmetric growth of the bilateral TMJ, reducing the height of the condyle on the affected side and resulting in a skewed mandible [29]. For adolescents in their growth spurt, early repositioning of the articular disc is recommended as soon as possible. This helps restore the developmental potential of the condyle, thus reducing the risk of future dental and maxillofacial deformities, such as mandibular retraction [30, 31].

In clinical treatment, clinicians often lack knowledge of the etiology and development of TMD, which can result in incorrect treatment protocols, such as orthodontic and orthognathic protocols, performed and temporomandibular joint instability, leading to erratic results, complications, and an increased risk of relapse. This paper reviews the treatment options for disc reduction, compares their advantages and disadvantages, and serves as a reference for future clinical treatment selection.

## 2. Materials and methods

### 2.1 Data selection

A search was conducted on PubMed and Web of Science for articles related to the keywords ((((TMJ) AND (Disc displacement)) AND (Treatment)) AND (Disc repositioning)) AND (MRI) published between 2004 and 2024. The flow diagram was constructed according to PRISMA 2020 guidelines [32]. Fig. 1 shows that the search resulted in 119 records. Seventeen duplicates were automatically excluded, and an additional 10 were manually removed. Ninety-two records were eliminated. Fifty-five records were excluded after reviewing the title and abstract, as they were determined irrelevant to the topic. Thirty-five reports were retrieved. Thirteen records were excluded for “Review article” (n = 4), “Irrelative to disc reposition” (n = 4), or “Condylar fracture” reasons (n = 5). Ultimately, 24 records were included in this review. The articles were imported into Endnote software (Endnote X9.3.2, Thomson Research Soft, New York, NY, USA) for citations and references. A summary of the efficacy of various treatments, the populations for which they are indicated, and their advantages and disadvantages were reviewed from the records of the past 20 years. Future research directions for the treatment of disc reduction are also discussed.

**Fig. 1.** Flow diagram of the review process.

### 2.2 Key information intended

Key information was obtained from meta-analyses, reviews, clinical follow-up, and case reports, including the pros and cons of various non-invasive and invasive treatments. The treatments discussed here were obtained from articles on PubMed and Web of Science databases, including literature meeting the requirements of 2.1. As shown in Fig. 2, this review includes treatment for articular disc repositioning, covering five directions and focuses on the study methodology, treatment protocol, sample size, follow-up duration, efficacy, and prognosis (clinical symptoms and imaging changes).

**Fig. 2.** Treatment method and content of concern.

## 3. Results and discussion

### 3.1 Conservative treatments

Conservative treatments include pharmacological therapy, physiotherapy, electrical modalities, psychological intervention, dry needling or acupuncture and minimally invasive injections [33]. Manipulative repositioning can improve clinical symptoms such as pain, clicking, and limited mouth opening. However, the success rate of repositioning the disc-condyle relationship remains relatively low, ranging from 9% to 23% [34]. Rady *et al*. [35] compared the use of botulinum toxin type A and low-level laser therapy with anterior repositioning appliances in ADDwR patients. The results revealed significant improvement in disc position and joint space, but the ARS group was better able to maintain the disc-condyle relationship in the correct position for a longer period. The primary objective of conservative treatments is to alleviate TMD by relieving pain and asymptomatic clicking and restoring mouth opening. However, long-term efficacy remains a concern, and there is a lack of evidence from studies on of disc-condyle repositioning with conservative treatments. Therefore, treatments should focus on repositioning the TMJ discs or combining other treatments with conservative treatments to promote more favorable outcomes in the short and long term.

#### 3.1.1 Functional splints

Functional splint therapy is a non-invasive, reversible, and cost-effective treatment option for TMD. The use of functional splints in the early stages of TMD is effective in relieving symptoms. This therapy allows displaced discs to slide back into their normal positions, promotes disc-condyle regeneration, and recapitulates the normal disc-condyle relationship by reattaching the condyle to the disc or reducing the pressure between the disc and the condyle [36].

The functional splints used mainly include ARSs, SSs, and other innovative splints. The ARS and SS can stabilize the disc-condyle relationship and cushion the occlusion preoperatively in TMJ surgery. This reduces the disc-condylar pressure and increases the space between the disc and the condyle, providing a solid foundation for TMJ surgery and maintaining the normal disc-condyle relationship postoperatively [37]. In addition, ARSs and SSs are comfortable to wear, increasing patient satisfaction and improving treatment effectiveness [38].

Table 1 (Ref. [5, 9, 21, 39, 40, 41, 42, 43]) presents the results of all functional treatment studies that met the inclusion criteria outlined in Section 2.1 and includes imaging results from 8 studies. The majority of patients who received functional therapy reported that short-term signs improved. However, whether patients can maintain a long-term, stable disc-condyle relationship and experience reduced recurrence after treatment has yet to be determined.

**Table 1.** Summary of retrospective analysis.

Serial number	Author	Diagnosis	Treatment method	Sample size	Age	Follow-up time	Imaging examination	Imaging result	Clinical symptom
1	Yano *et al*. [39]	ADDwR and ADDwoR	Splint therapy	82 joints, 41 patients	12 to 55 (mean: 23)	Not mentioned	MRI	Improved (29.3%), double contour (18.9%)	Not mentioned
2	Badel *et al*. [40]	ADDwR and ADDwoR	Michigan splint therapy	25 patients	18 to 71 (mean: 38)	5 mon	MRI	No change (92%)	Pain elimination (72%)
3	Ma *et al*. [41]	Skeletal Class II malocclusions and ADDwR	ARS	91 joints, 72 patients	Mean: 15.7	12 mon	MRI	Recapture with “double contour” (64.83%); disc recapture (92.31%—end) (72.53%—after 12 mon)	No TMJ clicking (89.1%—end), (86.6% —after 12 mon)
4	Shen *et al*. [42]	ADDwR	ARS	210 joints, 144 patients	9 to 53	6 to 16 mon	MRI	Successfully repositioned (84.3%—end) (84.8%—3 to 6 mon) (75.0%—7 to 12 mon) (72.1%—13 to 24 mon) (53.1%—>24 mon); Successfully repositioned (84.72%—<20 yr) (84.21%—21 to 35 yr) (77.78%—>36 yr)	Not mentioned
5	Wang *et al*. [43]	ADDwR	ARS	29 patients	Mean: 20.8 yr	3–6 mon	MRI	Effective disc displacement (95%); New bone formation (46%)	Improvement of joint clicking (97%), abnormal opening (77.8%) and joint pain (77.8%)
6	Di Paolo *et al*. [9]	ADDwoR	RA.DI.CA. Splint	10 patients	Mean: (41.10)	6 mon	MRI	Disc recaptured (70%)	Mandibular movement improved (70%)
7	Li *et al*. [21]	ADDwR	Step-back ARS retraction	48 adults	Mean: (27.1)	3 mon	MRI	Joint disc recaptured (92.1%); Bilaminar zone (31.25%)	Maximum interincisal opening increased mean 1 mm
8	Chen *et al*. [5]	ADDwR	ARS	22 subjects	Mean: (23.32)	6 mon	MRI	Normal disc-condyle relationships (100%—end); Normal disc-condyle relationship (40.6%—after 6 mon)	Joint clicking and intermittent locking disappeared (100%—end); Relapse of joint clicking and no pain (26.9%—after 6 mon)

*ADDwR: anterior disc displacement with reduction; ADDwoR: anterior disc displacement without reduction; ARS: anterior repositioning splint; SS: stabilization splint; MRI: Magnetic resonance imaging; TMJ: temporomandibular joint; RA.DI.CA.: Rampello–Di Paolo-Cascone.*

##### 3.1.1.1 Anterior repositioning splint

The mechanism of anterior repositioning splint (ARS) therapy is primarily attributed to disc-condyle relationship correction, improvement in stress distribution, and condyle remodeling [44]. It has an evident effect in the short term, but the long-term stability is not optimal; thus, applications should be selected carefully.

Wang *et al*. [43] reported the “double line sign” after ARS treatment via MRI. The treatment is effective for anterior and anterolateral displacement but not for anteromedial displacement. Ma *et al*. [41] reported that the ARS was relatively effective in repositioning the ADDwR, particularly in early adolescents. However, the success rate of recapture was lower in late adolescents, especially in patients over 16 years of age. Additionally, the efficacy of ARS treatment decreased over time. Shen *et al*. [42] also concluded that the ARS method is less effective in the long-term recapture of ADDwR.

##### 3.1.1.2 Stabilization splint

Liu *et al*. [45] conducted a comparative study on the use of the ARS and SS and reported that the ARS position significantly reduced postoperative disc displacement, facilitating complete disc repositioning. Additionally, the success rate of repositioning with the SS was notably lower than that with the ARS. However, Li *et al*. [46] surveyed ADD patients and reported that with the SS therapy, the disc-condyle relationship improved effectively. Korkmaz *et al*. [47] concluded that stabilization splints are an acceptably successful treatment modality for reducing clicking in the temporomandibular joint; however, this study did not address the disc-condyle relationship. Ding *et al*. [48] studied ADDwoR patients and reported that only 1 in 21-disc positions improved. This finding suggests that application of the SS may not be an effective approach for disc repositioning in patients with ADDwoR.

A stabilization splint can slightly increase the vertical occlusal distance. When there is occlusal contact in both planes of the plate, it can reduce the symptoms of ADDwoR [49, 50].

#####  3.1.1.3 Innovative splints

Di Paolo *et al*. [9] investigated the use of Rampello–Di Paolo-Cascone (RA.DI.CA). splints and found that most of the subjects’ discs recaptured and improved after treatment. This specialized splint facilitates anterior mandibular movement, allowing the condyle to move forward to recapture the articular disc. Patients reported improvements in headaches and neck pain after treatment, as well as improved mandibular opening. However, it may cause discomfort while being worn [9]. Additionally, Li *et al*. [21] conducted research on step-back anterior repositioning (SAR) splints. This method is appropriate for patients with deep overbite and overjet who have ADDwR. It has been demonstrated to enhance retrodiscal tissue adaptations and condylar bone remodeling.

#### 3.1.2 Comments on functional splints

In summary, patient age is closely related to the effect of condylar regeneration after disc repositioning. Therefore, studying the relationships between age and the impact of disc repositioning and improvement in clinical symptoms is essential for revealing the relationships between the growth and regeneration of the disc, the condylar structure and disc repositioning at different stages of development. In addition, untreated ADDwoR results in excessive anterior displacement of the disc over time. Although mandibular opening may improve, compensatory widening of the ligaments of the articular discs may exacerbate disc problems. Therefore, symptomatic follow-up alone has limited clinical relevance, and monitoring imaging changes combined with clinical symptom changes is essential. The condyle usually appears to have a double contour image after functional splints, but the mechanism behind this is currently unknown and may be due to splinting to restore the displaced disc to its normal position, allowing for regeneration in the condylar crest and posterior border area. By measuring the volume of the condyle via CT, we can more accurately assess bone formation, and the quantitative analysis allows a more accurate assessment of osteophytes, particularly in relation to age and different interventions. By combining clinical symptoms with imaging measurements using various imaging methods, we can evaluate anatomical and functional integrity and assess the therapeutic effectiveness [9].

The mechanism of splint therapy remains controversial. Although functional splints are a conservative, non-invasive, and reversible methods that relieve disc-condyle stress, most splints effectively alleviate pain, improve joint function, and reduce clicking syndrome. However, long-term stability is not optimal, possibly due to an unstable disc-condyle relationship, joint regeneration, occlusion or muscle system instability, or poor patient compliance. Therefore, disc repositioning procedures such as anchorage or arthroscopy should be promptly performed if conservative treatment is unsuccessful.

### 3.2 Anchorage

Roh *et al*. [51] reported that the incidence of condyle resorption was four times greater in patients with ADDwoR than in the general population. Condyle growth in teenagers can be affected by ADD. In untreated adolescent patients with ADDwoR, studies have shown that condylar height decreases [27, 52, 53, 54, 55]. According to Wolford and Cardenas [55] and other relevant studies [56], disc repositioning has been shown to alleviate condylar bone resorption and promote bone regeneration, particularly in adolescents. He *et al*. [57] proposed the open suturing (OSu) technique, which is based on the self-designed hook described in Yang’s arthroscopic surgery. This technique has a broader range of indications and is easier to perform in patients with hyperplastic posterior bands or perforations. When the disc and condyle connection is loose, a titanium plate and nail are used for anchoring, as they provide greater stability. However, wire anchoring can be used when the connection is more stable, such as in cases of mortise and tenon combination after a fracture and when the patient is younger and has a strong disc-condylar tissue regeneration ability [58].

Liu *et al*. [59] and Han *et al*. [60] reported that anchorage surgery relieves patients’ pain and increases mouth opening. MRI revealed that most of the disc-condyle relationships is stabilized after disc repositioning, with significant condylar regeneration. However, for patients with small condyles, due to conditions such as those with idiopathic condylar resorption and osteoporosis, the procedure of implanting the anchors is difficult. Furthermore, the blood supply around the condyle can also be affected, which increases the risk of condylar resorption [61]. Therefore, implementing artificial joint displacement, which is beyond the scope of this study, is recommended. Zhu *et al*. [54] investigated the effects of either mini-screw anchor (MsA) or OSu surgery on condylar regeneration and bony class II deformities in adolescent patients. Both treatments promoted condylar regeneration and improved bony class II deformity. Among these methods, OSu is associated with better postoperative outcomes, with greater bone regeneration in the condyle. Lu *et al*. [62] compared open suturing and mini-screw anchors and reported that most patients improved the disc position, and 62.5% of patients experienced improved condylar bone remodeling with both methods. Compared with mini-screw anchors, open suturing results in better disc position stability and condylar bone remodeling. Zhou *et al*. [63] concluded that open anchoring can be performed under open direct vision. This method is suitable for patients who are deep in the glenoid fossa, are older, have a small degree of tension, have perforated discs, and have osteoarthritis. Choi *et al*. [58] discussed the necessity of concurrent articular disc restoration in addition to simple open reduction and internal fixation (ORIF) surgery after condylar neck fractures, as complete symptomatic and imaging recovery cannot be achieved with ORIF surgery alone.

Currently, disc repositioning can be performed with wire or screw anchorage via arthroscopy or anchorage [57, 64]. Anchorage via screws is recommended for patients with short articular disc length and perforation, and the disc can cover the condyle after repositioning; however, the lack of an intermovement disc-condyle relationship during mandibular movement after repositioning requires relatively strong retention force. Anchorage using wire is indicated for patients with a normal condylar process or good bone quality and sufficient articular disc length and can be easily repositioned on the condyle [31]. Following repositioning, the condyle and disc drive each other during mandibular movement, causing translation and rotation of the joint [65]. The clinical efficacy of the two anchoring methods is comparable, with both methods providing reliable and stable anchors. However, the use of screws may result in artifacts in subsequent patient imaging and necessitate secondary surgical removal of the anchor [38]. On the other hand, anchorage by wire requires a strict indication and has a lower anchoring force. Nevertheless, it does not require removal and does not affect subsequent imaging.

### 3.3 Arthroscopy

With advancements in equipment and surgical techniques, TMJ arthroscopy has become a widely used tool for both diagnosis and treatment [66]. However, in the early days of the arthroscopy, the success rate was relatively low [51]. Arthroscopy is a highly effective procedure that not only relieves pain and restricts mouth opening but also prevents resorption and degeneration of the condyle and disc [67]. Furthermore, more than 70% of patients exhibit new bone formation after surgery [68]. Arthroscopy is a less invasive procedure that can be performed under local anesthesia and does not require postoperative drainage. Compared with anchorage, it has fewer postoperative complications and a shorter duration of hospitalization [69].

Yang *et al*. [70] and Liu *et al*. [19] reported a new arthroscopy method that can provide stable disc repositioning for more than 2 years in most patients. The modified method without anchor implantation renders it suitable for patients with hypoplastic condyles. Hu *et al*. [28] and Tang *et al*. [71] reported that cartilage growth in the condyle occurs where the articular disc covers the condyle. The remodeling process is a crucial indicator of how the joint adapts to altered biomechanics in the long term [72]. The application of finite element analysis has been demonstrated to effectively simulate the location of bone stress distribution in diverse disc-condyle relationships following arthroscopic surgery [73, 74]. Consequently, it is postulated that this technique can also be employed to quantify the location and stress of condylar remodelling after arthroscopic surgery of the temporomandibular joint. This may replace some clinical trials and assist clinicians in elucidating the biomechanical alterations associated with disparate treatments for their patients in a lucid and comprehensible manner.

To summarize, arthroscopy can move the disc from an anterior to an overcorrected position. The process of anchoring the disc backward and upward is the opposite of that achieved by the ARS, which repositions the articular disc by recapturing it with the condyle [75]. Arthroscopy repositions the disc-condyle relationship, thereby promoting new bone formation in the condyle and leading to morphological changes in the joint by pulling the disc posteriorly and superiorly. This movement allows the disc to move from a highly folded and shortened configuration to a more normal state, which may improve the disc’s coverage of the condyle [71]. This phenomenon occurs because of the repositioning of the disc-condyle relationship and disperses the mechanical stress on the condylar surface. As a result, the condyle undergoes bone remodeling, leading to a significant increase in volume.

Askar *et al*. [76] reported no significant differences in long-term clinical performance between arthroscopy and anchorage. Research conducted by He *et al*. [77] and Zhou *et al*. [63] concluded that anchorage is more traumatic than arthroscopy. However, the two procedures differ in their indications and patient acceptability. Arthroscopy is a minimally invasive procedure with small postoperative scars that can be performed under local anesthesia, making it suitable for repositioning the articular disc in patients who are not eligible for general anesthesia. However, it may lead to complications such as pain and increased restriction of mouth opening. The indications for arthroscopy are more stringent, requiring good elasticity and regeneration of TMJ tissues, a shallow glenoid fossa, good disc morphology, minimal deformation, no disc perforation, and proper mouth opening. However, in cases where the glenoid fossa is deep, impeding access to arthroscopic instruments, or in obese patients or those with a low degree of tumescence, the anchorage method is preferred for repositioning. Postoperatively, there was no significant difference in prognosis between the two treatments.

Furthermore, arthroscopic disc repositioning necessitates a range of equipment, including arthroscopes, light sources, monitors, coblation devices, and high-level technical expertise [70]. Fig. 3 compares arthroscopic and anchorage indications. Consequently, anchors are still used for disc repositioning when the required device and technique are unavailable, and when the indications are met [57].

**Fig. 3.** Indications between arthroscopy and anchorage.

### 3.4 Combined orthodontic and disc reposition

Due to the limitations of functional splints, combination therapy has been introduced to improve mandibular function, reposition the TMJ disc, promote new bone formation in the condyle, and maintain normal occlusion in adolescents. Capurso and Marini [78] conducted a study on the combined use of functional splints and orthodontic treatment and reported significant improvement in mandibular function in most patients. Many researchers have recently combined different treatment combinations to treat TMJ disc displacement. Liu *et al*. [79] investigated the use of arthroscopic disc repositioning surgery for treating unilateral mandibular retraction with three combined treatments: an anteriorly displaced splint, a two-block aligner, and a Herbst aligner. The results indicated that the combined treatment approach resulted in significant condylar height restoration and better outcomes in younger patients, which is consistent with the findings of Capurso and Marini [78].

Additionally, Sun *et al*. [31] reported that open suturing surgery combined with postoperative occlusal splints (POS) significantly promoted condylar neointegration in adolescents with Class II bone deformity. Ding *et al*. [48] examined occlusal stabilization splints with or without arthroscopic disc repositioning in adolescent patients with ADDwoR. They reported that the combined splint and arthroscopic treatment achieved better disc positioning with condyle bone generation. In patients with excessive pressure on the TMJ, alleviating the pressure between the disc and condyle using splints before surgery can achieve better surgical results [80]. Disc repositioning stabilises the disc-condyle relationship, increasing the stability of subsequent treatment. Conversely, orthodontic treatment before and after disc repositioning can also stabilize the disc-condyle relationship, providing a more stable and long-term effect.

As demonstrated above, disc repositioning and orthodontic treatments are mutually beneficial and closely intertwined. Patients who meet the surgical indications for arthroscopy and anchorage often present with severe inward tilt of the upper anterior teeth, abnormal inclination of the incisal guides or anterior underbite, narrow maxillary dental arches, and posterior teeth with locked and cross-locked bites. After simple articular disc repositioning, the likelihood of articular disc displacement recurring after surgery is high due to the lack of space for anterior mandibular displacement and occlusal interference. Therefore, presurgical orthodontic treatment is necessary before disc repositioning to correct occlusal malocclusion. Orthodontic treatment should correct the compensatory malocclusion caused by occlusal discrepancies and provide adequate space for surgery, thereby reducing recurrence. Aligning the dental arches before repositioning restores the normal occlusal relationship, ensuring stable long-term effects and reducing the recurrence of articular disc displacement.

### 3.5 Combined orthognathic and disc reposition

Fujimura *et al*. [81] conducted a study on patients with ADD treated with intraoral vertical ramus osteotomy (IVRO) and reported no significant improvements in the position of the articular disc. Sharma *et al*. [82] studied patients with skeletal class II malocclusion who underwent combined orthodontic and bilateral sagittal split ramus osteotomy (BSSRO). This study revealed a small degree of disc repositioning in ADDwR patients but no significant clinical changes in ADDwoR patients. Gonçalves *et al*. [83] followed patients with orthognathic surgery complexes with or without an ADD. Their results showed that patients who underwent simultaneous TMJ disc repositioning had a stable postoperative jaw position. On the other hand, patients who did not undergo TMJ disc repositioning were prone to a recurrence in the postoperative period. Wolford *et al*. [84] reported that patients who underwent double-jaw surgery with a preoperative ADD experienced condylar resorption with Class II open bite malocclusion. Conversely, Goncalves *et al*. [85] reported that patients who underwent simultaneous articular disc repositioning during bimaxillary surgical advancement presented at least 1.5 mm of postoperative bone deposits, whereas those without resurfacing presented no bone deposits. Therefore, orthognathic treatment based on an unstable disc-condyle relationship may exacerbate joint symptoms and fail to improve the disc-condyle relationship. Several studies have shown that surgical correction of maxillofacial deformities while repositioning and stabilizing the articular discs, can provide high-quality outcomes in most patients [83, 84, 85].

The efficacy of combined treatment is contingent upon the growth potential of the adolescent patient. Determining whether to perform orthopedic treatment or orthognathic surgery is challenging, particularly when the sole criterion is the patient’s age and no consensus exists among clinicians [86]. Emission computed tomography (ECT) technology can measure the bone growth potential of patients with malocclusion, enabling earlier intervention for those undergoing orthognathic surgery. On the other hand, it is unnecessary to intervene in the disc position in patients who have already stabilized growth and have little potential for bone gain. Furthermore, the process of condylar reconstruction may be affected by the joint space and condylar cartilage pressure [79].

On the other hand, not all patients require presurgical disc repositioning if the disc-condyle relationship is stable. Correct anatomical structures and the absence of severe deformities characterize the stable relationship. Shen *et al*. [7] reported that articular disc repositioning is not necessary in stable cases without clinical symptoms, including those who have completed growth and development, have an intact condylar cortex, have no articular degeneration, have articular disc-like changes in the bilaminar zone, and have no changes in the articular and maxillofacial clinical and imaging manifestations at an interval of 6 months or more. Therefore, older patients with low bone growth potential and displaced articular discs but no obvious clinical symptoms, orthodontic and orthognathic procedures can be performed, even with deformed but stable disc-condyle relationships, improving facial shape and maintaining long-term stability.

Furthermore, for patients with Class II malocclusion, treatment or surgery involving disc repositioning can be employed to improve the condition to a certain extent [31, 54]. This involves condylar cartilage regeneration and anterior and inferior displacement of the condyle after anchoring and guiding the mandible to move forward as a whole, thus improving the Class II facial shape. In some cases, continuing orthodontic or orthognathic treatment may be unnecessary if the disc-condyle relationship remains stable. This can be achieved in a less invasive manner, thus meeting the patient’s aesthetic requirements.

## 4. Conclusion

Conservative treatment can effectively improve joint symptoms but is ineffective in repositioning displaced discs, and long-term therapeutic effects need to be discussed in further studies. Arthroscopic or anchorage to reposition the articular disc effectively improves disc displacement and serves as the stabilizing cornerstone for further orthodontic and orthognathic treatments. Postoperative use of functional splints, with orthodontic and orthognathic treatment, can further promote condylar osteogenesis, improve facial shape, and prevent recurrence. However, there is a lack of research examining the long-term effects of treatment, the influence of early intervention for adolescent disc displacement on facial development, and digital technologies such as finite element analysis in TMJ treatment. Consequently, there is a need for more high-quality research in these areas.
